# The Anaphase-Promoting Complex Protein 5 (AnapC5) Associates with A20 and Inhibits IL-17-Mediated Signal Transduction

**DOI:** 10.1371/journal.pone.0070168

**Published:** 2013-07-29

**Authors:** Allen W. Ho, Abhishek V. Garg, Leticia Monin, Michelle R. Simpson-Abelson, Lauren Kinner, Sarah L. Gaffen

**Affiliations:** 1 Division of Rheumatology and Clinical Immunology, University of Pittsburgh, Pittsburgh, Pennsylvania, United States of America; 2 Department of Oral Biology, School of Dental Medicine, University at Buffalo, State University of New York, Buffalo, New York, United States of America; Karmanos Cancer Institute, United States of America

## Abstract

IL-17 is the founding member of a family of cytokines and receptors with unique structures and signaling properties. IL-17 is the signature cytokine of Th17 cells, a relatively new T cell population that promotes inflammation in settings of infection and autoimmunity. Despite advances in understanding Th17 cells, mechanisms of IL-17-mediated signal transduction are less well defined. IL-17 signaling requires contributions from two receptor subunits, IL-17RA and IL-17RC. Mutants of IL-17RC lacking the cytoplasmic domain are nonfunctional, indicating that IL-17RC provides essential but poorly understood signaling contributions to IL-17-mediated signaling. To better understand the role of IL-17RC in signaling, we performed a yeast 2-hybrid screen to identify novel proteins associated with the IL-17RC cytoplasmic tail. One of the most frequent candidates was the anaphase promoting complex protein 7 (APC7 or AnapC7), which interacted with both IL-17RC and IL-17RA. Knockdown of AnapC7 by siRNA silencing exerted no detectable impact on IL-17 signaling. However, AnapC5, which associates with AnapC7, was also able to bind IL-17RA and IL-17RC. Moreover, AnapC5 silencing enhanced IL-17-induced gene expression, suggesting an inhibitory activity. Strikingly, AnapC5 also associated with A20 (*TNFAIP3*), a recently-identified negative feedback regulator of IL-17 signal transduction. IL-17 signaling was not impacted by knockdown of Itch or TAXBP1, scaffolding proteins that mediate A20 inhibition in the TNFα and IL-1 signaling pathways. These data suggest a model in which AnapC5, rather than TAX1BP1 and Itch, is a novel adaptor and negative regulator of IL-17 signaling pathways.

## Introduction

Interleukin-17 (also known as IL-17A, referred to hereafter as IL-17) is the hallmark cytokine of Th17 cells, a relatively new CD4+ T cell subset [1], [2]. Th17 cells also produce IL-17F, a closely related member of the IL-17 cytokine family, a heterodimer of IL-17A/F, and IL-22 [3], [4], [5]. Th17 cells and IL-17 cytokines coordinate the adaptive and innate immune systems to promote inflammatory reactions. Perturbations in the IL-17/Th17 axis are linked to defects in host defense against pathogens, but also to the development of autoimmune diseases such as rheumatoid arthritis (RA), multiple sclerosis and psoriasis [6]. Antibodies against IL-17 or its receptor have shown exciting promise in recent clinical trials to treat several autoimmune conditions [7], [8], [9], [10].

Despite advances in understanding Th17 cell biology, mechanisms that govern IL-17-mediated signal transduction remain less well understood. The IL-17R is part of a novel subfamily of cytokine receptors that is strikingly distinct in sequence and structure from other receptors [11], [12]. IL-17, IL-17A/F and IL-17F all signal via a multimeric receptor complex composed of two subunits, IL-17RA and IL-17RC [13], [14], [15], [16]. In addition, IL-17RA partners with other members of the IL-17R family to form distinct receptor complexes, including IL-17RB (to form the IL-25 receptor) and IL-17RE (creating the IL-17C receptor) [17], [18], [19], [20]. Thus far, IL-17RC is not known to participate in other complexes and therefore appears to be a unique receptor for the IL-17A, A/F and F cytokines [16], [21].

Members of the IL-17 receptor family contain a conserved domain within the cytoplasmic tail known as a “SEFIR” for SEF/IL-17R conserved motif. The SEFIR is not found in other receptor families, but bears some homology to the Toll like receptor/IL-1R (TIR) domain found in the TLR/IL-1 receptor families [22], [23]. The SEFIR serves as a platform for recruitment of proximal signaling intermediates. Although the TIR-containing adaptor MyD88 is not required for IL-17R signaling [24], Act1 (also known as Connection to IKK and SAP/JNK, CIKS) contains a SEFIR and binds to IL-17RA and IL-17RC via their respective SEFIR domains [25], [26]. Act1 is both an adaptor and an E3 ubiquitin ligase that recruits and activates downstream TNF receptor associated factors (TRAFs) [27], [28], [29]. Act1 recruits TRAF6, also an E3 ubiquitin ligase, which leads to activation of the NF-κB, MAPK (mitogen activated protein kinase) and CCAAT/Enhancer Binding Protein (C/EBP) pathways, which in turn regulate expression of inflammatory genes encoding cytokines, chemokines and antimicrobial proteins [30], [31]. Alternatively, recruitment of TRAF5 and TRAF2 to Act1 promotes stability of target gene mRNA transcripts, which is particularly well documented for the chemokine CXCL1 (KC, Groα) [32], [33].

IL-17-mediated signaling is generally quite modest compared to more potent inflammatory mediators such as TNFα or lipopolysaccharide (LPS), but it synergizes potently with TNFα, lymphotoxin-β and other cytokines [34]. The IL-17 signaling pathway is kept in check by a variety of downstream inhibitors, which help limit collateral damage in settings of acute infection. To list a few examples, Act1 is inducibly degraded following receptor stimulation by the SCFβ-TrCP (Skp1, Cul1, F-box protein β-TRCP) complex [35]. The transcription factor C/EBPβ is induced via glycogen synthase kinase 3β (GSK3β) and can inhibit expression of certain IL-17 target genes, particularly those regulated positively by C/EBPδ [36]. MiR-23b blocks IL-17 signaling by targeting the NF-κB pathway and IκBζ, and is associated with several autoimmune diseases [37]. NF-κB activation is dampened by reversing ubiquitination, and in this regard the ubiquitin specific peptidase 25 (USP25) and A20 deubiquitinases have both been recently implicated in restricting the IL-17 pathway [38], [39]. While USP25 activity appears to be specific to the TLR and IL-17 pathways [38], [40], A20 restricts NF-κB activation by TNFα, IL-1, Nod-like receptors and TLRs as well as IL-17 [41].

Efforts to decipher signaling events downstream of the IL-17 receptor have largely focused on the IL-17RA subunit. However, IL-17RC confers essential but poorly understood signaling properties [21]. Mutants of IL-17RC lacking the cytoplasmic tail, especially the SEFIR domain, fail to rescue signaling in an IL-17RC-deficient setting [13], [42]. Accordingly, in this study we undertook a screen to identify proteins associated with the IL-17RC cytoplasmic domain. One of the most frequent candidates was the anaphase promoting complex protein 7 (AnapC7, also known as APC7), which co-immunoprecipitated with both IL-17RC and IL-17RA. By itself, AnapC7 showed no detectable impact on IL-17 signaling. However, AnapC5, which has considerable sequence and functional homology to AnapC7 [43], was also able to bind IL-17RA and IL-17RC, and AnapC5 silencing enhanced IL-17-induced gene expression, suggesting an inhibitory activity. Strikingly, AnapC5 could also bind to A20 and interact with IL-17RA in the same receptor subdomain that binds to A20 [39]. In the TNFα and IL-1 pathways, A20 normally associates with the accessory proteins including Itch and Tax-binding protein 1 (TAXBP1) to mediate inhibition of signaling [41], [44], but we found that neither of these proteins appeared to participate in the IL-17 pathway. Therefore, these data suggest a new model in which AnapC5 participates in IL-17 signal transduction by means of complexing with the inhibitor A20.

## Materials and Methods

### Cell Culture and Mice

Mice were housed in specific pathogen-free conditions at the animal facility of the University of Pittsburgh. Tail biopsies were obtained following euthanasia, with all efforts to minimize suffering. The University of Pittsburgh Institutional Animal Care and Use Committee (IACUC) approved all animal protocols used in this study (Animal welfare assurance number: A3187-01). IL-17RC^−/−^, Rrad^−/−^, and CFD^−/−^ fibroblasts were isolated from IL-17RC^−/−^, Rrad^−/−^, and CFD^−/−^ tail biopsies [45], [46], [47]. ST2 cells [48], Human embryonic kidney (HEK) 293T cells (ATCC) and all fibroblast lines were cultured in α-MEM (Sigma, St. Louis, MO) with 10% FBS (Gemini BioProducts, Sacramento CA), L-Glutamine, and antibiotics (Invitrogen, Carlsbad, CA). HEK293T cells were transfected with CaPO_4_. Within each transfection experiment, 3 replicate samples were transfected and assayed separately. Cytokines were from Peprotech (Rocky Hill, NJ) and used at 200 ng/mL (IL-17A, IL-17F) or TNFα (2 ng/ml). IL-17RC−/− mice were provided by Genentech, in collaboration between Genentech and Lexicon Pharmaceuticals. Mice were handled in accordance with approved University of Pittsburgh IACUC protocols.

### Plasmids

IL-17RC and IL-17RA constructs were described previously [42], [49]. AnapC7 and AnapC5 cDNA constructs were generated by RT-PCR from ST2 cells and expressed in the pCMV4 vector fused to 3′ Myc or HA tags. All constructs were confirmed by sequencing.

### Yeast Two Hybrid Screening

A custom tetracycline repressor (TetR) based system was used for screening (ProteinLinks, Pasadena CA) [50]. Briefly, the bait DNA construct contains the murine IL-17RC cytoplasmic tail fused to the TetR. ProteinLinks transformed the construct into yeast cells containing a library of prey proteins from a mouse 3T3 library fused to a transcriptional activation domain.

### siRNA Knockdown and Qpcr

Specific siRNAs (Thermo Scientific, Waltham, MA) for the target genes (Anapc5, Anapc7, Act1, A20, TAX1BP1, ITCH, USP25, scrambled control) were transfected with DharmaFECT reagents according to manufacturer’s instructions. ST2 cells were seeded overnight in antibiotic-free medium and transfected the next day with 50 nM siRNA with DharmaFECT Reagent 1 (Dharmacon/Thermo Scientific). 24 hours later, culture medium was changed, and after a further 24 hours cells were stimulated with IL-17. For each experiment, 3 replicate samples were analyzed separately. For qPCR, total RNA was isolated using RNeasy mini-columns (Qiagen, Valencia, CA). cDNA was prepared from 2 mg of oligo-dT primed total RNA using the ThermoScript reverse transcription system (Invitrogen, Carlsbad, CA). Real-time PCR was performed using a 7300 Real-time PCR System (Applied Biosystems, Foster City, CA) and PerfeCTa SYBR Green FastMix, ROX (Quanta Biosciences, Foster City, CA). For quantification, target genes were normalized to GAPDH. For each experiment, 3 replicates were analyzed separately.

### Western Blotting, Immunoprecipitation and ELISA

Western blotting and immunoprecipitation were performed as described [49], [51]. Anti-Myc and anti-A20 Abs were from Cell Signaling (Cell Signaling, Beverly, MA). HA Abs were from Roche. Protein molecular weights were determined using the Fermentas Prestained Protein Ladder (Thermo Scientific). IL-6 protein levels were determined using the IL-6 ELISA Ready-SET-Go kit (eBioscience, San Diego, CA). For each experiment, each sample was analyzed in duplicate or triplicate, and a minimum of 3 replicates were included per experiment.

### Statistics

To assess statistical significance, we used Student’s t-Test (for two comparisons) or ANOVA with post-hoc Tukey’s analysis (for more than two comparisons in an experiment). *P<0.05 was considered significant. Error bars reflect the means+SEM of biological replicates within individual experiments. Chi square analysis was used to determine whether the qualitative changes between replicate experiments were statistically reproducible. All experiments were repeated a minimum of twice to ensure reproducibility.

## Results

### Yeast Two-hybrid Screen to Identify IL-17RC-associated Proteins

Because the IL-17RC cytoplasmic tail is essential for IL-17-dependent signaling [13], [42], we hypothesized that unidentified proteins associate with this subunit and contribute to IL-17-mediated signal transduction. Accordingly, we fused the murine IL-17RC cytoplasmic domain (amino acids 484–698) to the DNA binding domain of the tetracycline repressor to serve as “bait” in a yeast two hybrid genetic screen (ProteinLinks, see **Methods**). The IL-17RC-tetR bait was screened against an NIH3T3 library expressing genes fused to the B42 transactivation domain [50], yielding 11 candidates (**Table 1**). One of the most frequent candidates was 3-hydroxyisobutyrate dehydrogenase; however, since housekeeping genes are common yeast-two-hybrid false positives, this gene was not considered further. Other candidates encoded hypothetical proteins with no known function, and were also not analyzed subsequently. Three candidates had potential roles in biochemical and/or immune signaling and hence were pursued in this study: Ras-related associated with diabetes (Rrad), complement factor D (CFD, also known as adipsin) and anaphase promoting complex subunit 7 (AnapC7).

**Table 1:** Yeast Two-Hybrid Screen of IL-17RC cytoplasmic domain.

*Accession ID*	*Number of hits*	*Description*
BC026808.1	11	Hypothetical protein LOC76824; part of DUF279 superfamily
BC003914.1	10	3-hydroxyisobutyrate dehydrogenase
NM_026633	7	Hypothetical protein LOC68241; no conserved domains
AK159523.1	7	Anaphase promoting complex subunit 7
BC147795.1	2	Centromere protein V
NM_025623.2	1	Nipsnap homolog 3A
NM_019662.2	1	Rrad; Ras like GTPase superfamily member
M11768.1	1	Complement Factor D (adipsin); trypsin-like serine protease
AL670091.26	1	Unknown
FJ374665.1	1	Unknown
AC215630.1	1	Unknown

Results of screen performed by ProteinLinks. Number of positive hits, accession number and description of gene product are indicated.

### There is no Apparent Role for Rrad or CFD in IL-17 Signaling

**Figure 1:**
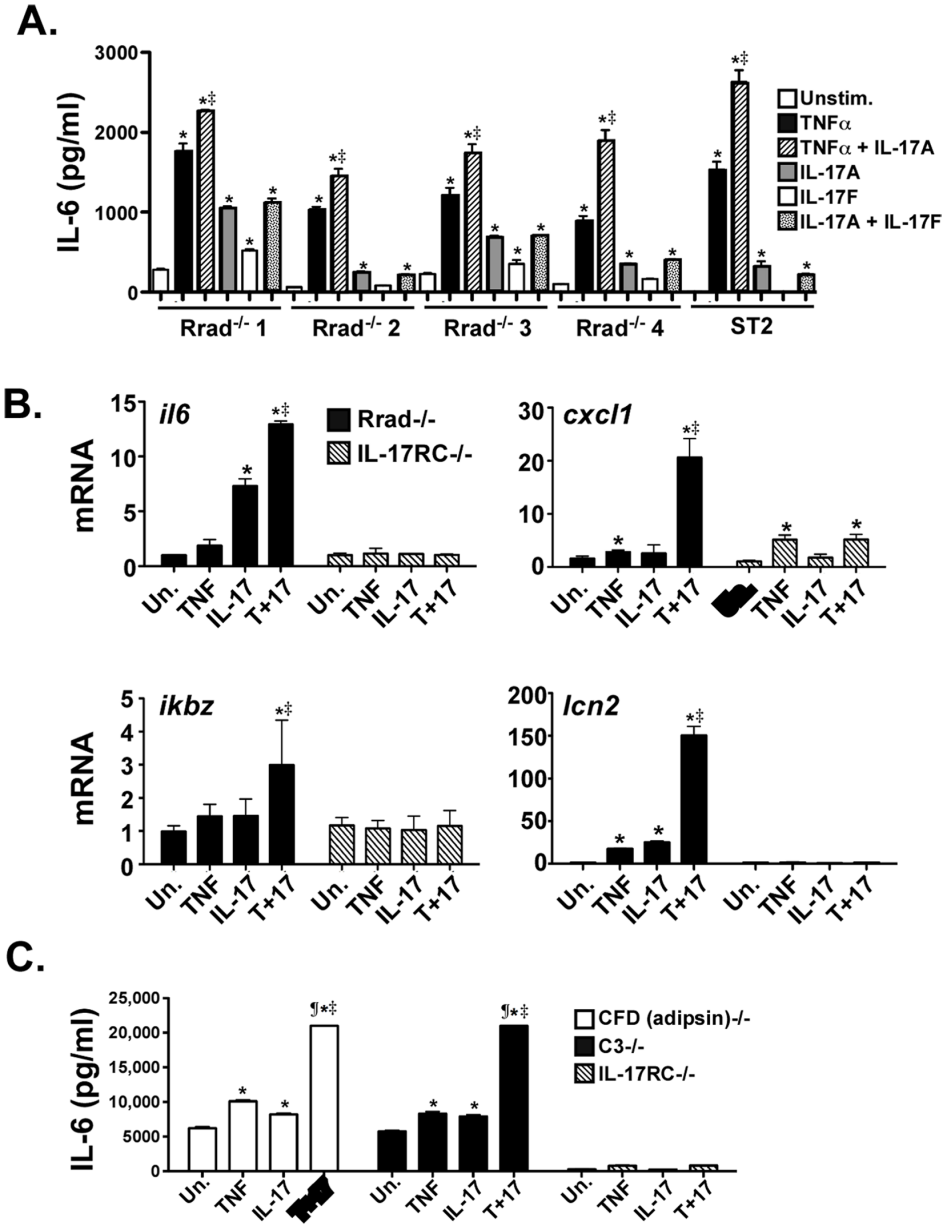
Neither Rrad nor CFD mediate IL-17 signal transduction. A. Rrad-deficient fibroblasts mediate normal IL-17 induction of IL-6 secretion. Multiple Rrad^−/−^ cell lines (derived from adult tail biopsies of Rrad^−/−^ mice) or ST2 stromal cell lines were treated with IL-17 (200 /ng/ml), IL-17F (200/ ng/ml) or suboptimal TNFα (2 ng/ml) for 24 h. IL-6 was measured in culture supernatants in triplicate by ELISA. *p<0.05 by ANOVA and post-hoc Tukey’s test compared to unstimulated controls. ^‡^ *p*<0.05 by Chi Square comparing experimental replicates. B. Rrad-deficient fibroblasts mediate normal IL-17 induction of target gene expression. A representative Rrad^−/−^ cell line or IL-17RC^−/−^ fibroblasts were treated with IL-17 or TNFα as outlined in panel A for 24 h. Expression of the indicated genes was assessed by qPCR in triplicate. *p<0.05 by ANOVA and post-hoc Tukey’s test compared to unstimulated controls of each cell line. ^‡^ *p*<0.05 by Chi Square comparing experimental replicates. C. Complement deficient cell lines mediate normal IL-17 signaling. Fibroblast cell lines from CFD^−/−^, C3^−/−^ or IL-17RC^−/−^ mice were treated with IL-17 and TNFα as described in panel A, and IL-6 concentrations in culture supernatants were assessed by ELISA. *p<0.05 by ANOVA and post-hoc Tukey’s test compared to unstimulated controls. ^‡^ *p*<0.05 by Chi Square comparing experimental replicates. ^¶^concentration values above standard curve for ELISA detection. All data are representative of at least two independent experiments.

Rrad is the prototypic member of a subfamily of Ras-related small G proteins originally identified in patients with Type 2 diabetes [52]. Notably, Rrad mRNA was downregulated in IL-17RA^−/−^ mice in a microarray screen comparing tissue in WT or IL-17RA-deficient animals infected with the yeast *Candida albicans* [53], thus linking Rrad to the IL-17 pathway. To investigate whether Rrad functionally mediates IL-17-dependent signaling, we created primary fibroblast cell lines from Rrad^−/−^ mouse tail biopsies [46]. We verified that cells expressed the IL-17RA and IL-17RC by flow cytometry (data not shown). Rrad^−/−^ fibroblasts were capable of mediating signaling, as they produced IL-6 in response to IL-17 alone or in synergy with TNFα, as shown by ELISA of conditioned supernatants **(** **Fig. 1A** **)**. Consistently, IL-17± TNFα induced expression of several well-described IL-17 target gene targets, including *lcn2* (encoding 24p3, also known as lipocalin 2), *cxcl1* (CXCL1, Groα, KC), *ikbz* (IκBζ), and *il6* (IL-6) **(** **Fig. 1B** **)** [6]. Some, though not all cell lines showed responsiveness to IL-17F; however, this cytokine exhibits extremely modest activity, and it is often difficult to elicit any detectable signals with this cytokine. Since at least half the lines responded to IL-17F, we conclude that Rrad is not required to mediate IL-17- or IL-17F-mediated signaling.

Complement factor D (CFD, also known as adipsin) is a serine protease that regulates the alternative complement pathway by cleaving Factor B when it is bound to C3, initiating formation of the C3 convertase. Factor B represents the only currently known CFD target [54]. To determine whether CFD plays a role in IL-17 signaling, we prepared fibroblast lines from tail biopsies of CFD^−/−^ mice [47]. As shown, CFD^−/−^ fibroblasts responded to IL-17 alone or in synergy with TNFα by producing IL-6 at levels similar compared to C3^−/−^ control fibroblasts **(** **Fig. 1C** **)**. Thus, CFD does not appear to contribute to IL-17-dependent signaling.

### AnapC7 Associates with the IL-17 Receptor Subunits but is Dispensable for IL-17-Dependent Signaling

**Figure 2:**
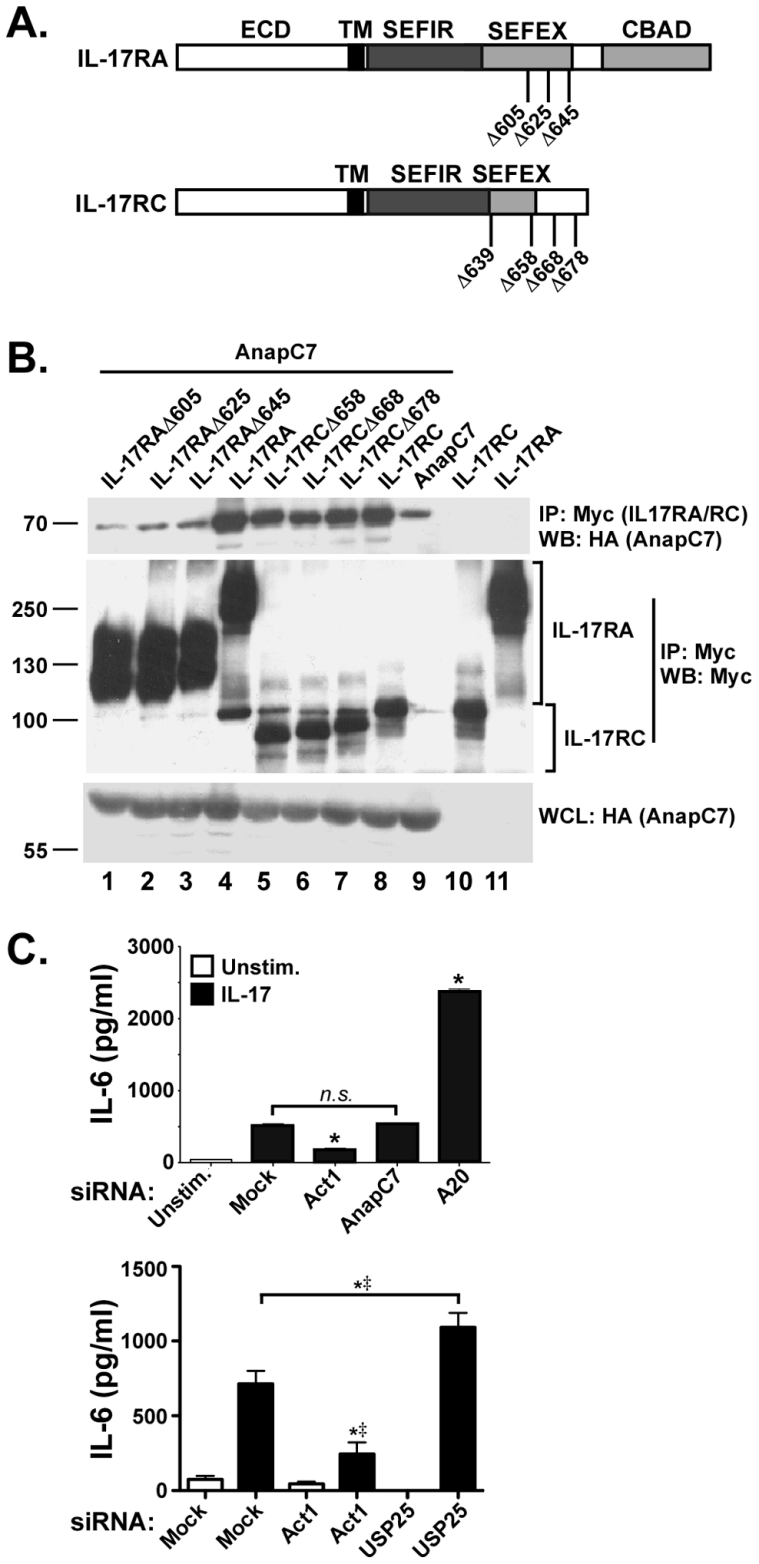
AnapC7 binds to IL-17R but does not impact IL-17 signaling. **A. Schematic diagram of IL-17RA and IL-17RC mutants.** ECD, extracellular domain. SEFIR and SEFEX domain approximate boundaries are indicated [22], [24]. CBAD, C/EBPβ activation domain. **B. AnapC7 associates with IL-17RA and IL-17RC.** HEK293T cells were transfected with AnapC7 tagged with HA and IL-17RA or IL-17RC tagged with Myc, as indicated. Lysates were immunoprecipitated with anti-Myc Abs and immunoblotted for HA or Myc. Whole cell lysates (WCL) were verified for AnapC7 by staining with anti-HA. Migration of protein size markers is indicated. **C. RNA silencing of AnapC7 does not alter IL-17-dependent signaling.** ST2 cells were transfected with the indicated siRNAs, treated with IL-17 (black bars) for 24 h and IL-6 in culture supernatants assessed by ELISA. *n.s.,* not significant. *p<0.05 by ANOVA and post-hoc Tukey’s test relative to unstimulated controls. ^‡^ *p*<0.05 by Chi Square comparing experimental replicates.

AnapC7 is a vertebrate-specific subunit of the anaphase promoting complex/cyclosome (APC/C), a multi-protein E3 ubiquitin ligase required for eukaryotic mitotic progression [43]. AnapC7 itself does not possess enzymatic function, and has been shown to interact with the coactivators Creb-binding protein (CBP) and p300 and stimulate their transcriptional activity [55]. To determine whether AnapC7 associated with IL-17RC or other IL-17R subunits, HEK293T cells were co-transfected with AnapC7 (HA-tagged) together with full length IL-17RA and IL-17RC (Myc-tagged) or a panel of receptor truncation mutants, and their ability to interact was evaluated by co-immunoprecipitation. AnapC7 associated with IL-17RC, confirming the yeast two hybrid result. AnapC7 associated with IL-17RC mutants truncated at the C-terminal end of the SEFIR domain **(** **Fig. 2A–B** **)**, suggesting that the association of AnapC7 with IL-17RC occurs somewhere within the conserved SEFIR domain. AnapC7 also associated with full-length IL-17RA **(** **Fig. 2A** **)**. However, a deletions of IL-17RA lacking the C-terninal “CBAD” domain abrogated this association. Thus, AnapC7 interacts with both IL-17RA and IL-17RC.

There are no available AnapC7 knockout mice. To determine whether AnapC7 plays a role in IL-17 signaling, we silenced AnapC7 via siRNA knockdown in ST-2 cells, an IL-17-responsive stromal cell line. Knockdown of AnapC7 had no impact on IL-17-dependent IL-6 production at the mRNA or protein levels **(** **Fig. 2C** **)**. As controls, we verified that siRNA silencing of Act1, a positive mediator of IL-17 signaling, reduced IL-17 signaling as measured by IL-6 production. As expected, silencing of the deubiquitinases (DUB) A20 or USP25, recently shown to be inhibitors of IL-17 signaling [38], [39], resulted in enhanced IL-17-mediated IL-6 production (**Fig. 2C**). These results demonstrated that, while AnapC7 associates efficiently with the IL-17 receptor, its activity is dispensable for functional IL-17-mediated signaling, either positively or negatively.

### AnapC5 Negatively Regulates IL-17 Receptor Signaling

**Figure 3:**
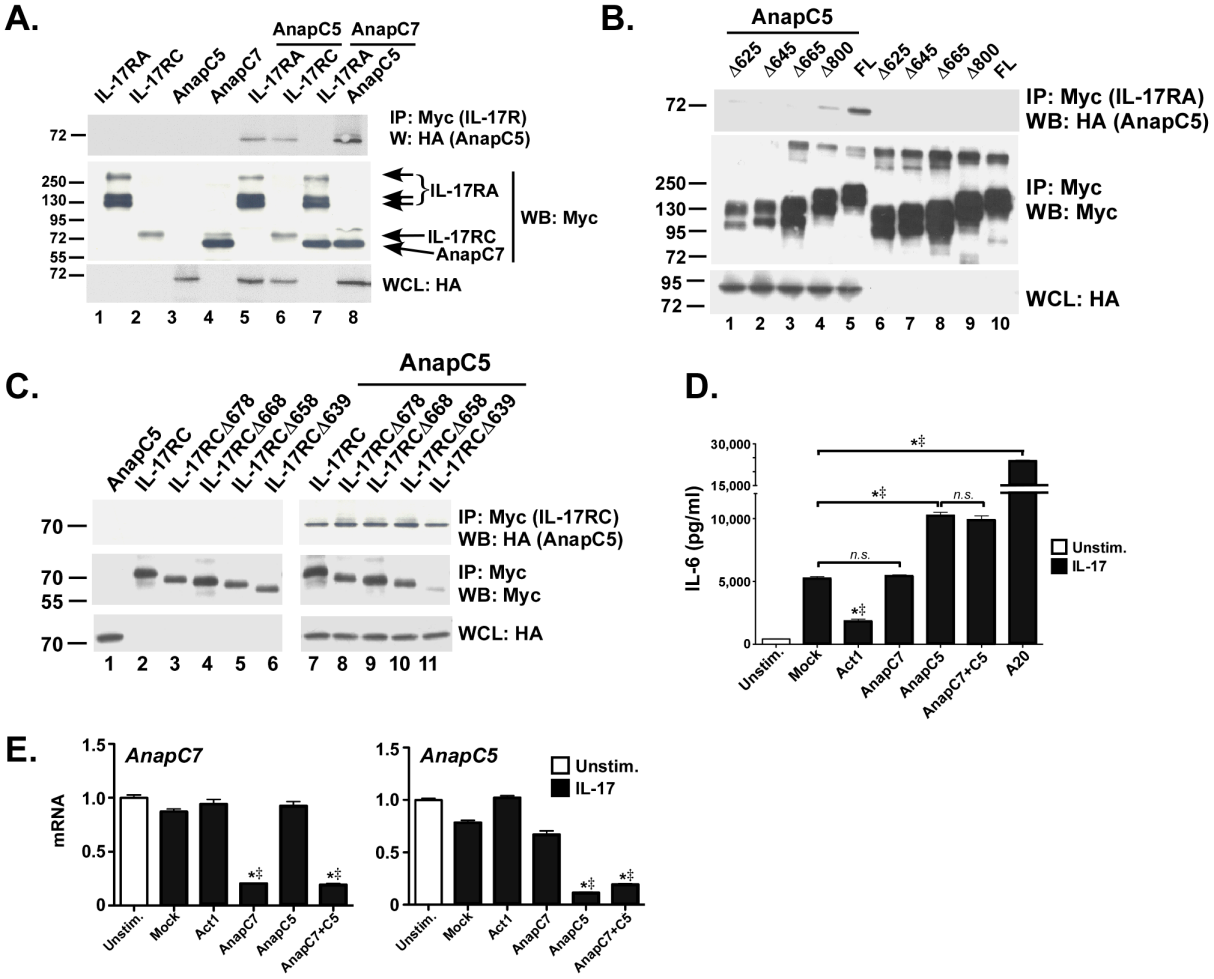
AnapC5 binds to the IL-17 receptor and restricts IL-17-mediated signal transduction. A. AnapC5 associates with IL-17RA, IL-17RC and AnapC7. HEK293T cells were transfected with AnapC5 (tagged with HA) together with IL-17RA, IL-17RC or AnapC7 (tagged with Myc), as indicated. Lysates were subjected to IP with anti-Myc Abs and immunoblotted with anti-HA or anti-Myc. Arrows indicate identity of each Myc-tagged protein. Whole cell lysates (WCL) were verified for AnapC7 by staining with anti-HA. Migration of protein size markers is indicated. B. AnapC5 associates with IL-17RA through the inhibitory CBAD domain. HEK293T cells were transfected with AnapC5 and the indicated IL-17RA deletion constructs. Lysates were subjected to co-IP with anti-Myc and blotted for HA or Myc. Whole cell lysates (WCL) were verified for AnapC7 by staining with anti-Myc. Migration of protein size markers is indicated. C. AnapC5 associates with IL-17RC in the SEFIR domain. HEK293T cells were transfected with AnapC5 and the indicated IL-17RC deletion constructs. Lysates were subjected to co-IP with anti-Myc and blotted for HA or Myc. Whole cell lysates (WCL) were verified for AnapC7 by staining with anti-HA. Migration of protein size markers is indicated. D. Knockdown of AnapC5 enhances IL-17 signaling. ST2 cells were transfected with the indicated siRNAs, stimulated with IL-17 for 24 h, and IL-6 in culture supernatants assessed by ELISA. *n.s.,* not significant. *p<0.05 by ANOVA and post-hoc Tukey’s test compared to unstimulated controls. ^‡^ *p*<0.05 by Chi Square comparing experimental replicates. E. Efficient knockdown of AnapC5 and AnapC7. mRNA from the samples in panel D were assessed for AnapC5 and AnapC7 expression by qPCR. *p<0.05 by ANOVA and post-hoc Tukey’s test compared to unstimulated controls. ^‡^ *p*<0.05 by Chi Square comparing experimental replicates. Data are representative of at least 2 independent experiments.

AnapC5 is another structural component of the APC/C. Like AnapC7, AnapC5 also activates the CBP/p300 complex [55]. Because of the analogous roles played by AnapC5 and AnapC7 in CBP/p300 regulation and also the presence of common structural motifs in both proteins, we hypothesized that AnapC5 might participate in IL-17 signaling. To determine whether AnapC5 associated with the IL-17 receptor, HEK293T cells were co-transfected with AnapC5 and IL-17RA or IL-17RC, and interactions between these factors were evaluated by co-IP. AnapC5 associated efficiently with IL-17RA and IL-17RC (**Fig. 3A**, lanes 5–6). We further verified that AnapC5 also associated with AnapC7 (**Fig. 3A** **,** lane 8). Thus, both AnapC5 and AnapC7 form a complex with the IL-17 receptor.

We then sought to delineate motifs within the IL-17R required for interaction with AnapC5. HEK293T cells were co-transfected with AnapC5 and a panel of IL-17RA truncation mutants. AnapC5 associated only with full-length IL-17RA and to a lesser extent IL-17RAΔ800, but not with other deletion mutants despite equivalent expression of the receptor mutants **(** **Fig. 3B** **)**. Thus, AnapC5 associates with a C-terminal domain of IL-17RA. This domain corresponds to the C/EBPβ-activation domain (CBAD) in IL-17RA [49], [56], which was previously linked to inhibitory signaling [24], [36], [39], [57]. We also attempted to define regions within the IL-17RC cytoplasmic tail necessary for co-association using a panel of IL-17RC truncation mutants. AnapC5 co-immunoprecipitated with all IL-17RC deletion mutants tested, suggesting that the site of interaction is within the SEFIR domain **(** **Fig. 3C** **)**.

To determine whether the association of AnapC5 with IL-17RA/C impacted IL-17 signaling, we employed siRNA to silence AnapC5 in ST2 cells **(** **Fig. 3D** **)**. When compared to cells transfected with a control siRNA, knockdown of AnapC5 resulted in significantly increased IL-17-dependent IL-6 expression (**Fig. 3D** **)**, suggesting that AnapC5 exerts an inhibitory function. Similar findings were made for *il6* mRNA (data not shown). Silencing efficiency was verified by qPCR (**Fig. 3E**). These findings indicated that AnapC5 binds to the IL-17R and dampens IL-17-mediated signal transduction. These data are consistent with the binding of AnapC5 to the CBAD, which is associated with negative regulation of IL-17 signaling (see **Discussion**).

### AnapC5 Associates with A20 Through the CBAD Motif in IL-17RA

**Figure 4:**
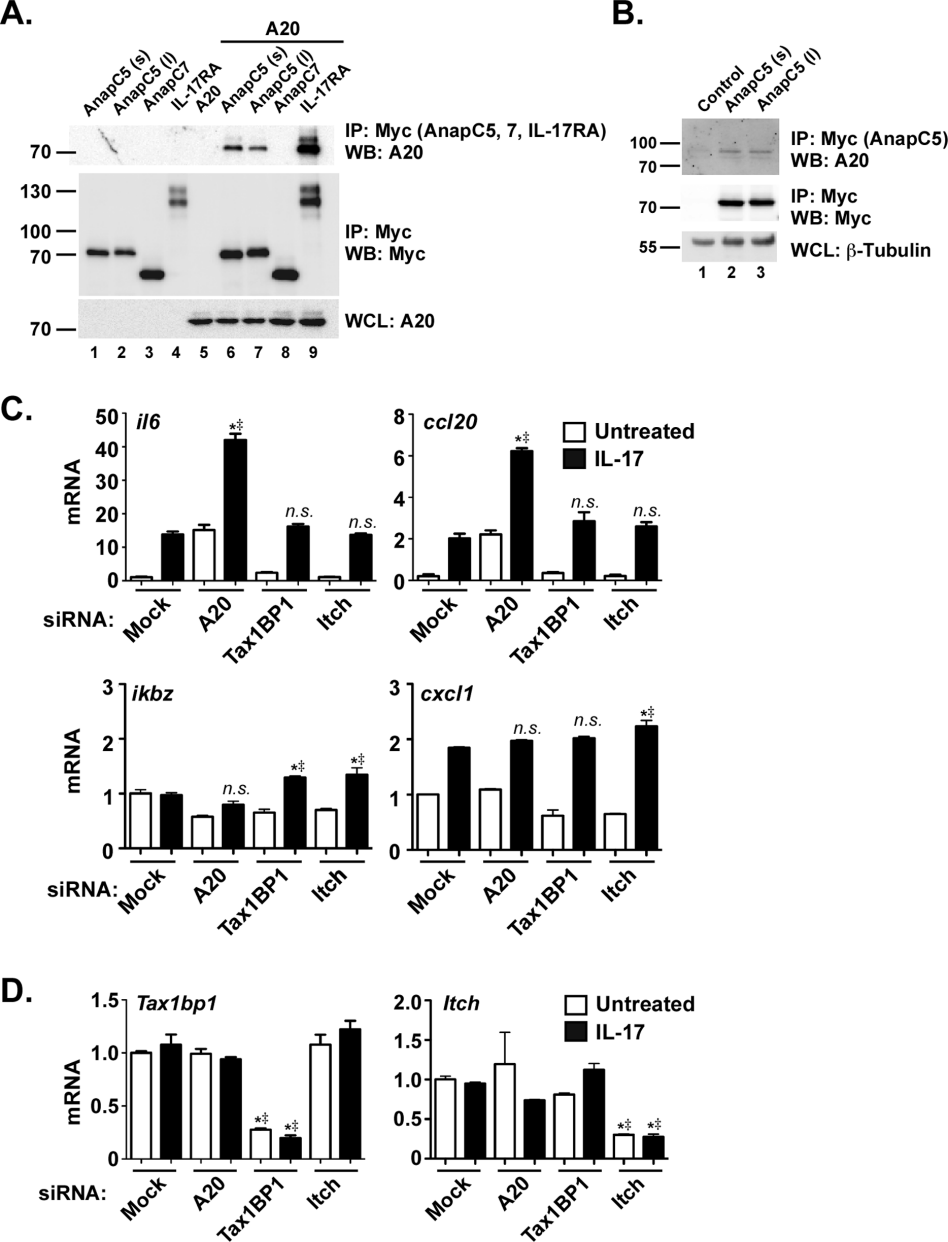
The IL-17R-A20 signaling complex involves AnapC5 rather than Itch and TAXBP1. A. AnapC5 but not AnapC7 associates with A20. HEK293T cells were co-transfected with AnapC5 [short (s) and long (l) forms] or AnapC7 (both Myc-tagged) together with A20. Lysates were subjected to IP with anti-Myc Abs and blotted for A20 or Myc. WCL were blotted for A20. Migration of protein size markers is indicated. B. AnapC5 associates with endogenous A20. HEK293T cells were transfected with Myc-tagged AnapC5. Lysates were subjected to IP with anti-Myc Abs and blotted for A20 or Myc. WCL were blotted for β-tubulin as a loading control. C. Knockdown of Itch and TAXBP1 does not impact IL-17R mediated signaling. ST2 cells were transfected with the indicated siRNAs, stimulated with IL-17 for 24 h, and mRNA expression was assessed by qPCR. *n.s.,* not significant. *p<0.05 by ANOVA and post-hoc Tukey’s test compared to unstimulated controls. ^‡^ *p*<0.05 by Chi Square comparing experimental replicates. D. Efficient knockdown of Itch and TAXBP1. mRNA from the samples in panel B were assessed for Itch and TAXBP1 expression by qPCR. *p<0.05 by ANOVA and post-hoc Tukey’s test compared to mock-transfected controls. ^‡^ *p*<0.05 by Chi Square comparing experimental replicates. All data are representative of at least two independent experiments.

**Figure 5:**
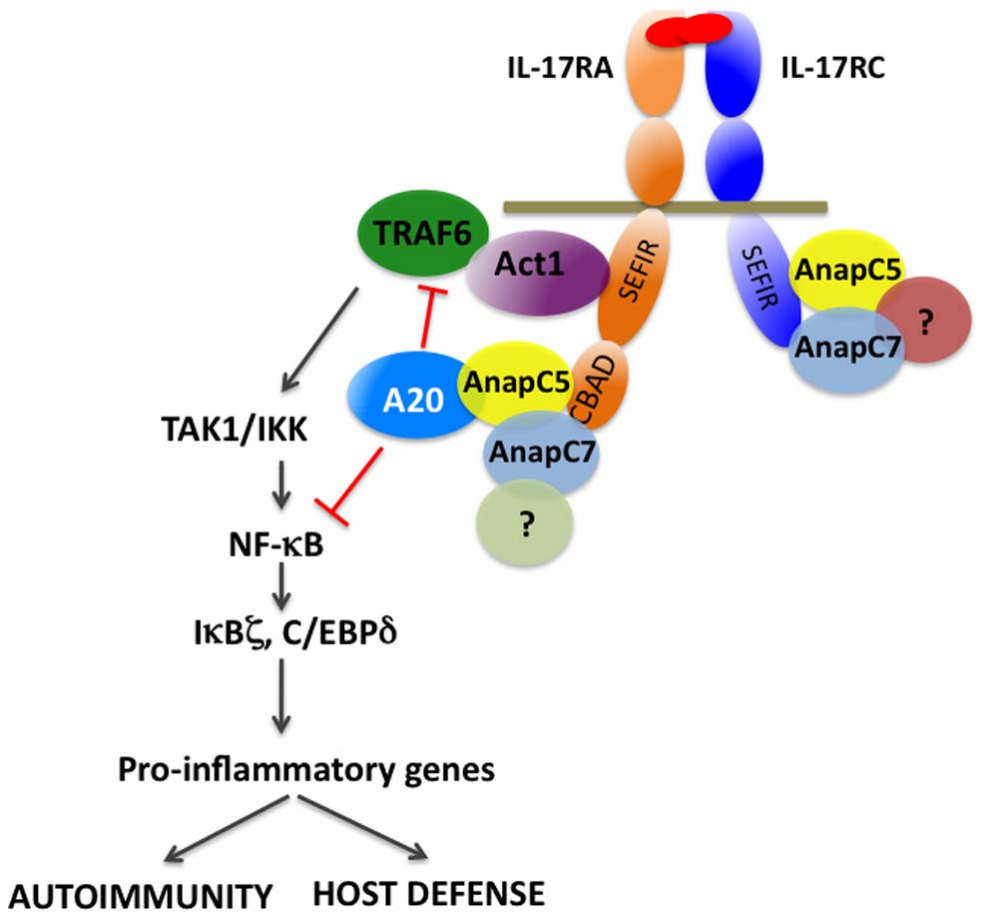
Schematic model of AnapC5 and AnapC7 in IL-17R signaling. The data in this paper support a model in which AnapC5 serves an adaptor or scaffold protein to facilitate A20 recruitment to the CBAD domain of IL-17RA. Although AnapC7 binds to both IL-17RA and IL-17RC, its functional role is still unclear.

Our recent data implicated the DUB A20 as a negative regulator of IL-17-dependent signaling, which binds to the CBAD within IL-17RA [24], [36], [57], [58]. Since AnapC5 associated with the CBAD and knockdown of AnapC5 enhanced IL-17 signaling, we hypothesized that AnapC5 might interact with A20. To test this hypothesis, A20 was co-transfected with AnapC5 (both the long and short forms of the gene) and AnapC7. As shown, A20 indeed co-immunoprecipitated with AnapC5, but not with AnapC7 **(** **Fig. 4A** **)**, potentially explaining the failure of AnapC7 knockdown to impact IL-17 signaling. Thus, AnapC5 associates with A20 and may facilitate the inhibitory functions of A20 downstream of the IL-17R (**Fig. 5**). To verify this finding in a more physiological setting, we transfected HEK293T cells with AnapC5 constructs and saw co-immunoprecipitation of endogenous A20, while there was no precipitation with a control plasmid (**Fig. 4B**).

### The A20 Accessory Molecules Itch and TAXBP1 are not Involved in Inhibitory IL-17 Signaling

Accessory molecules such as TAX1BP1 and Itch associate with A20 to form an active inhibitory complex in the TNFα and IL-1/TLR signaling cascades [44]. Given the negative regulatory role of A20 in the IL-17 signaling pathway and the potential of AnapC5 to function as an A20 accessory molecule, we investigated whether TAX1BP1 and Itch were required for IL-17-mediated inhibitory signaling. Surprisingly, knockdown of TAX1BP1 and Itch expression with siRNA revealed no significant modulation in IL-17-dependent expression of *il6* or *ccl20* mRNA levels as measured by qPCR**.** Similar findings were made for additional IL-17 target genes including *ikbz* and *cxcl1,* though we observed very modest enhancements in these genes upon knockdown (**Fig. 4C** **)**. The efficiency of TAX1BP1 and Itch knockdown was high, as confirmed by qPCR **(** **Fig. 4D** **)**. These data suggest that the A20 accessory molecules TAX1BP1 and Itch are dispensable for the negative regulatory functions of A20 downstream of IL-17, and that AnapC5 may instead serve in this capacity.

## Discussion

IL-17 has emerged as a critical inflammatory cytokine involved in both host defense and autoimmune pathology. IL-17 promotes disease in a variety of mouse models of multiple sclerosis, RA and inflammatory bowel disease [59], [60], [61], [62]. Consequently, the IL-17 family is an appealing biological target that shows efficacy in early clinical trials [63]. On the host side, IL-17 is important for immunity to extracellular pathogens, most strikingly those of fungal origin such as *Candida albicans* [64], [65].

Efforts to understand the IL-17 signaling pathway have lagged behind other cytokine families. Superficially, IL-17 appears to share many of the same pathways and gene targets as other pro-inflammatory cytokines, engaging the NF-κB and MAPK pathways and inducing expression of canonical inflammatory mediators such as IL-6 and CXC chemokines [2], [11]. However, IL-17R structure and signaling have proven to be atypical in multiple ways. The three-dimensional architecture of IL-17 family receptor and ligands are distinct from other cytokines [14], [66], [67]. The proximal adaptor Act1, required for NF-κB signaling, is not employed as a positive signaling intermediate by any other known system [27]. Similarly, the means by which IL-17 promotes stability of target mRNA transcripts is unusual, involving the Splicing Factor 2 RNA binding protein rather than the more classical tristetraprolin protein [32], [68].

The original goal of this study was to identify novel IL-17RC-interacting proteins in order to gain insights into how this receptor mediates downstream signal transduction. Surprisingly, Act1, the only protein known previously to bind IL-17RC, was not identified in this screen. Rrad and complement factor D appeared initially to be the most promising candidates, as they are involved in signal transduction and immunity (**Table 1**). However, cells from mice deficient in those genes signaled normally in response to IL-17 (**Fig. 1**). Although this does not absolutely rule out a role for either factor in the IL-17 signaling pathway, they do not seem to be critical players.

We turned our attention to AnapC7, one of the most frequent “hits” yielded in the two hybrid screen (**Table 1**). AnapC7 is best understood as a component of a macromolecular E3 ubiquitin ligase complex required for mitotic progression [43]. AnapC7 is also implicated in regulating gene transcription by interacting with the CBP/p300 coactivator and stimulating its acetyl transferase activity [55]. Although AnapC7 interacted with IL-17RA and IL-17RC, knockdown exerted no impact on IL-17 signaling, positively or negatively (**Fig. 2**). On the other hand, knockdown of AnapC5 enhanced IL-17 dependent signaling, suggesting it might function as an inhibitor of the pathway (**Fig. 3** **)**. Knockdown of another member of the APC complex, AnapC3, did not enhance IL-17-mediated signaling (data not shown). AnapC7 and AnapC5 share sequence homology with E1A, an adenoviral protein that drives cellular transformation via CPB/p300 complexes. The present work is to our knowledge the first evidence implicating AnapC5 as a signaling intermediate for cytokine signaling.

Polymorphisms in the gene encoding A20 (*TNFAIP3*) are implicated in various forms of autoimmunity and some cancers [41], [69]. A20 is a deubiquitinase that serves as a negative feedback inhibitor of TNFα, IL-1, and recently IL-17 [39], [41]. A20 acts on the IL-17 signaling pathway by down-modulating the NF-κB and MAPK pathways through deubiquitination of TRAF6 [39], [70]. In addition to its activity as a DUB, A20 also exhibits Ub ligase activity [71], which helps dictate the types of Ub linkages used to modulate signaling. A20 binds to IL-17RA through a distal inhibitory domain in the receptor known as the CBAD, a domain originally identified based on its role in regulation of the C/EBPβ transcription factor [24], [36]. Since AnapC5 is part of a multi-protein E3 Ub ligase complex and also binds to the CBAD, we speculated that its ability to inhibit IL-17 signaling might be regulated through A20. Indeed, A20 interacted strongly with AnapC5, but interestingly not with AnapC7 (**Fig. 4A, B**). Moreover, the A20-accessory proteins Itch and TAXBP1 had no apparent requirement in inhibiting IL-17-mediated signaling (**Fig. 4C**). Together, these data raised the possibility that the AnapC5 is part of a novel inhibitory complex that includes A20 and that assembles on the IL-17R CBAD domain (**Fig. 5**).

It is unclear what role, if any, is played by AnapC7 in IL-17 signaling. Unlike AnapC5, silencing of AnapC7 did not impact IL-17 induction of genes such as IL-6. One possibility is that AnapC7 acts as a scaffold protein for as-yet unidentified factors that mediate signal transduction specificity by IL-17RC (**Fig. 5**). In this regard, the role of the IL-17RC cytoplasmic tail in IL-17R signaling is still unresolved. Deletion studies indicate that the IL-17RC tail is essential [13], [42], so the receptor is evidently needed for more than just engaging the ligand. We previously showed that a dimer of the IL-17RC tail is insufficient to mediate signaling; namely, a chimeric receptor composed of the extracellular and transmembrane domain of IL-17RA fused to the cytoplasmic tail of IL-17RC could not rescue signaling in IL-17RA-deficient cells [58]. However, it is not known whether IL-17RC recruits novel proteins into the complex, or whether this subunit is simply needed to multimerize or stabilize existing signaling complexes by providing additional binding sites for proteins such as Act1 or the AnapCs.

There are important questions raised by this report that will need to be explored in future studies. For example, what are the relative contributions of A20, SCFβ/TrCP1 and USP25 on inhibition of IL-17 signaling? Are they redundant, cooperative or do they play tissue-specific roles? Does AnapC5 serve as a “switch” to favor one inhibitory pathway over the other? How important is the interaction of AnapC5 in binding to IL-17RC versus IL-17RA? Ultimately, defining the molecular events in the IL-17R pathway may yield new therapeutic targets, and will certainly provide new insights into how this intriguing cytokine receptor family operates.
